# Mitochondrial Transplantation Ameliorates Pulmonary Fibrosis by Suppressing Myofibroblast Activation

**DOI:** 10.3390/ijms252312783

**Published:** 2024-11-28

**Authors:** Seo-Eun Lee, Shin-Hye Yu, In-Hyeon Kim, Young Cheol Kang, Yujin Kim, Jeong Seon Yeo, Jun Hyeok Lim, Iksun Kwon, Je-Hein Kim, Se-Woong Park, Mi-Yoon Chang, Kyuboem Han, Sung-Hwan Kim, Chun-Hyung Kim

**Affiliations:** 1 Paean Biotechnology, Inc., 5 Samil-daero 8-gil, Jung-gu, Seoul 04552, Republic of Korea; selee0207@paeanbio.com (S.-E.L.); sryu2@paeanbio.com (S.-H.Y.); kangyc@paeanbio.com (Y.C.K.); yjkim@paeanbio.com (Y.K.); jsyeo@paeanbio.com (J.S.Y.); wnsgur109@paeanbio.com (J.H.L.); iksun0213@paeanbio.com (I.K.); kbhan@paeanbio.com (K.H.); 2 Graduate School of Biomedical Science and Engineering, Hanyang University, Seoul 04763, Republic of Korea; mychang@hanyang.ac.kr; 3 Division of Jeonbuk Advanced Bio Research, Korea Institute of Toxicology, Jeongeup 56212, Republic of Korea; inhyeon.kim@kitox.re.kr (I.-H.K.); jehein@kitox.re.kr (J.-H.K.); sewoong.park@kitox.re.kr (S.-W.P.); 4 College of Veterinary Medicine, Chonnam National University, Gwangju 61186, Republic of Korea; 5 Department of Premedicine, College of Medicine, Hanyang University, Seoul 04763, Republic of Korea

**Keywords:** idiopathic pulmonary fibrosis (IPF), stem cell, mitochondria, transplantation, anti-inflammation, antiapoptosis

## Abstract

Idiopathic pulmonary fibrosis (IPF) is a pulmonary disease characterized by excessive extracellular matrix protein deposition in the lung interstitium, subsequently causing respiratory failure. IPF still has a high medical unmet requirement due to the lack of effective treatments to inhibit disease progression. The etiology of IPF remains unclear, but mitochondrial dysfunction is considered to be associated with IPF development. Therefore, targeting mitochondrial abnormalities would be a promising strategy for treating IPF. Recently, exogenous mitochondrial transplantation has been beneficial for treating mitochondrial dysfunction. The current study aimed to examine the therapeutic effect of mitochondrial transplantation on IPF in vitro and in vivo. Mitochondria were isolated from human umbilical cord mesenchymal stem cells, referred to as PN-101. Human lung fibroblasts and human bronchial epithelial cells were exposed to transforming growth factor-β, followed by PN-101 treatment to determine the in vitro efficacy of mitochondrial transplantation. An IPF mouse model established by a single intratracheal instillation of bleomycin was utilized to determine the in vivo efficacy of the intravenously treated mitochondria. PN-101 attenuated mitochondrial damage, inhibited EMC production, and suppressed epithelial-to-mesenchymal transition in vitro. Additionally, intravenous PN-101 administration alleviated bleomycin-induced fibrotic processes in the IPF mouse model with a therapeutic context. Our data indicate that PN-101 is a novel and potential therapeutic agent for IPF.

## 1. Introduction

Idiopathic pulmonary fibrosis (IPF) is a debilitating pulmonary disease characterized by the excessive deposition of extracellular matrix (ECM) proteins, including collagen and fibronectin, in the lung interstitium, subsequently causing respiratory failure [1]. The etiology and pathophysiology underlying IPF remain unknown. Two approved anti-fibrotic drugs, namely pirfenidone and nintedanib, have slowed disease progression and increased the length of life for patients with IPF [2,3]. However, the mortality rate of patients with IPF is high, and the mean survival time has been estimated to be approximately 4 years [4]. Accordingly, IPF still has a high medical unmet need due to the lack of effective treatments to halt disease progression.

Mitochondria are critical organelles that maintain cellular physiology and survival by providing ATP as cellular energy. Besides ATP, mitochondria exhibit an important role in basic cellular functions, including ROS generation and scavenging, calcium ion homeostasis, signaling transduction, inflammation, and apoptosis [5,6]. Growing evidence indicates that mitochondrial abnormalities and metabolic dysregulation are present in the lung tissues of patients with IPF and are involved in IPF initiation and progression [7,8,9,10,11]. Abnormal mitochondrial respiration and oxygen consumption were found in the lung tissue of patients with IPF. Notably, reduced ATP production, decreased electron transport chain complex I and IV activity, and declined expression of mitochondria-encoded oxidative phosphorylation (OXPHOS) genes have been observed in lung fibroblasts, type II alveolar epithelial cells (AECII), and alveolar macrophages from the IPF lung, respectively [7,11]. Additionally, mitochondrial ROS are increased in the fibroblasts, AECII cells, and macrophages of lung tissue from patients with IPF as well as in bleomycin-induced IPF lungs of mice [8]. Together, maladaptation to cellular stress caused by mitochondrial damage results in IPF development. Therefore, targeting mitochondrial abnormalities is a promising strategy for treating IPF.

Recently, mitochondrial transplantation therapy, also known as mitotherapy, has become an attractive therapeutic strategy for diseases associated with mitochondrial dysfunction [12,13]. This novel therapy is according to the evidence that mitochondria isolated from various cell types could be efficiently taken up by cells or tissues. Transplantation of autologous, allogeneic, or xenogeneic mitochondria has exhibited therapeutic benefits in various animal models for human diseases, including ischemia/reperfusion (IR)-induced injury, tissue injury, and neurodegenerative disorders [14,15,16,17,18,19,20]. The pathophysiological benefits of mitochondrial transfer remain unclear, but the transfer of healthy mitochondria into cells of tissues with damaged mitochondria may improve a variety of cellular functions, including mitochondria biogenesis, oxidative stress, mitochondrial quality control, the inflammatory response, and apoptosis [21].

Recently, we developed clinical good manufacturing practice (GMP)-graded mitochondria, named PN-101, that were isolated from human umbilical cord mesenchymal stem cells (UC-MSCs). PN-101 demonstrated a potent anti-inflammatory effect by blocking the NFκB signaling pathway [22]. Intravenously injected PN-101 preferentially migrated to the lungs of bleomycin-induced IPF mice compared with the lungs of normal mice [23]. Importantly, a phase 1/2a clinical study (clinical trial number: NCT04976140) indicated that GMP-graded PN-101 exhibited good safety and efficacy in patients with refractory polymyositis and dermatomyositis.

The present study aimed to investigate PN-101 as a potential therapy for IPF. The results of the IPF in vitro model indicated that PN-101 attenuated mitochondrial damage, inhibited extracellular matrix protein (EMC) deposition, and suppressed epithelial-to-mesenchymal transition (EMT) in transforming growth factor-β (TGF-β)-treated human lung fibroblasts and epithelial cells. Additionally, intravenous PN-101 administration alleviated the fibrotic processes induced by the intratracheal instillation of bleomycin (BLM) in mice. Together, our data indicate that PN-101 can be a novel and potential therapeutic agent for IPF.

## 2. Results

### 2.1. Mitochondrial Transplantation Ameliorated Mitochondrial Damage in TGF-β-Treated Pulmonary Cells

**Figure 1:**
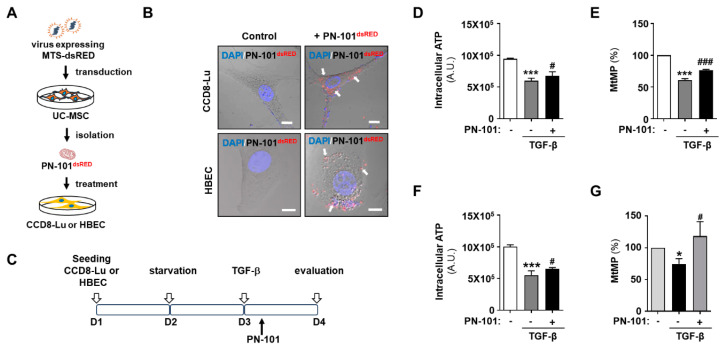
Exogenous mitochondrial transfer to human lung cells and reversal of TGF-β-induced mitochondrial dysfunction. (**A**) Schematic diagram of PN-101^dsRED^ treatment into human lung fibroblasts (CCD8-Lu) and human bronchial epithelial cells (HBECs). PN-101^dsRED^ was obtained from UC-MSCs, which were infected with a lentivirus expressing MTS-dsRED. (**B**) Representative immunofluorescence images. The red colored dots indicate the exogenously transferred PN-101^dsRED^. The cell nuclei were stained with DAPI. PN-101^dsRED^ was successfully transferred into the CCD8-Lu cells and HBECs (white arrows). The scale bar indicates 10 μm. (**C**) Schematic representation of PN-101 treatment in the TGF-β-induced fibrosis model. CCD8-Lu cells and HBECs were seeded, starved in a culture medium without FBS for 24 h, and then treated with TGF-β at 2.5 ng/mL. After 6 h, various PN-101 concentrations were administered for 24 h. Intracellular ATP and mitochondrial membrane potential (MtMP) were measured in CCD8-Lu cells (**D**,**E**) and HBECs (**F**,**G**), respectively. PN-101 treatment significantly attenuated the TGF-β-induced decrease in ATP and MtMP levels. All data are presented as mean ± SEM. * *p* < 0.05 and *** *p* < 0.001 versus control and ^#^ *p* < 0.05 and ^###^ *p* < 0.001 vs. PN-101 non-treatment.

Exogenous mitochondria enter any cell type in vitro via macropinocytosis [24,25,26,27]; thus, we first tested whether fluorescence-labeled PN-101 could be transferred to lung fibroblasts and bronchial epithelial cells. Hence, PN-101^dsRED^ were prepared from UC-MSCs infected with lentivirus-mediated MTS-dsRED and were co-incubated with CCD8-Lu cells and HBECs (Figure 1A). The confocal images demonstrated that PN-101^dsRED^ was exclusively localized to the cytoplasm, indicating the successful internalization of PN-101^dsRED^ into both cells (Figure 1B).

TGF-β plays a pivotal role in fibrosis development by promoting EMT induction [28], and its activation is associated with mitochondrial dysfunction in the lung [29]. Intracellular ATP level and mitochondrial membrane potential (MtMP) were assessed with or without PN-101 in CCD8-Lu cells and HBECs to investigate the effect of TGF-β on mitochondrial function in lung fibroblasts and epithelial cells (Figure 1C). Treatment of CCD8-Lu cells and HBECs with TGF-β, compared to controls, significantly caused a 37% and 45% reduction in ATP levels and 40% and 39% in MtMP, respectively (Figure 1D–G). Interestingly, PN-101 treatment notably attenuated the TGF-β-induced decrease in ATP and MtMP levels (Figure 1D–G). These results indicated that mitochondrial transplantation attenuated TGF-β-induced mitochondrial damage in pulmonary cells.

### 2.2. Mitochondrial Transplantation Reduced the Expression of Fibrotic Genes in TGF-β-Treated Lung Fibroblasts

**Figure 2:**
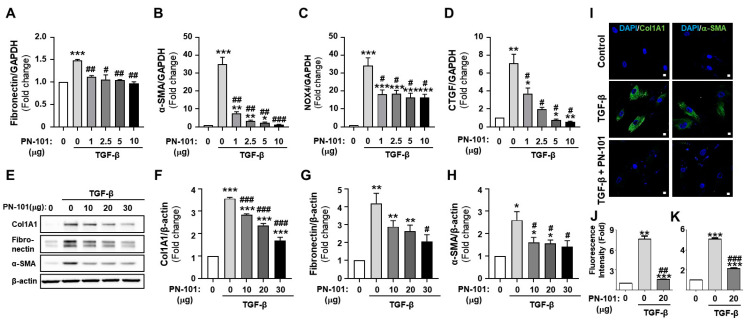
Anti-fibrotic effects of mitochondrial transplantation in the TGF-β-induced CCD8-Lu cells. Human lung fibroblasts, CCD8-Lu cells, were treated with TGF-β for 6 h after PN-101 administration for 24 h. (**A**–**D**) The mRNA expression of fibrotic genes was measured with quantitative RT-PCR. PN-101 treatment attenuated the mRNA expression of fibronectin, α-SMA, NOX4, and CTGF in TGF-β-stimulated CCD8-Lu cells dose-dependently. (**E**–**H**) Collagen type 1 A1 (Col1A1), fibronectin, and α-SMA protein levels were measured with a Western blot assay. PN-101 attenuated Col1A1, fibronectin, and α-SMA protein expression in TGF-β-stimulated CCD8-Lu cells in a concentration-dependent manner. (**I**) The Col1A1 and α-SMA proteins were highly expressed in TGF-β-stimulated CCD8-Lu cells as measured using an immunocytochemistry assay, whereas PN-101 abolished the TGF-β-stimulated expression of the Col1A1 (**J**) and α -SMA proteins (**K**). The scale bar indicates 10 μm. Data are presented as the mean ± SEM (n = 3). * *p* < 0.05, ** *p* < 0.01, and *** *p* < 0.001 vs. control. ^#^ *p* < 0.05, ^##^ *p* < 0.01, and ^###^ *p* < 0.001 vs. PN-101 non-treatment.

During lung fibrosis, TGF-β signaling promotes fibroblast transformation to myofibroblasts, where large amounts of ECM, including collagen and fibronectin, are deposited in the fibrotic region, aggravating PF [30]. We measured the fibrotic gene expression of the TGF-β-treated CCD8-Lu cells, followed by PN-101 treatment, to investigate the effect of mitochondrial transplantation on fibrotic progression in lung fibroblasts. First, the expressions of fibronectin, α-SMA, NADPH oxidase 4 (NOX4), and connective tissue growth factor (CTGF) mRNA were assessed with quantitative real-time polymerase chain reaction (qRT-PCR). Expectedly, the mRNA expression of all fibrotic genes was greatly increased in the TGF-β-treated CCD8-Lu cells. However, PN-101 treatment significantly alleviated the TGF-β-stimulated mRNA expression of fibrotic genes in CCD8-Lu cells dose-dependently (Figure 2A–D). We then measured the protein expression of collagen, type1, alpha1 (Col1A1), fibronectin, and α-SMA of the TGF-β-treated CCD8-Lu cells using a Western blot assay. The expression of Col1A1, fibronectin, and α-SMA were dramatically increased when CCD8-Lu cells were treated with TGF-β. However, PN-101 remarkably attenuated the TGF-β-induced increase in protein expression concentration independently (Figure 2E–H). Furthermore, the immunocytochemistry assay demonstrated that PN-101 treatment decreased the TGF-β-induced Col1A1 and α-SMA protein expressions in CCD8-Lu cells (Figure 2I–K). Together, these results strongly indicated that mitochondrial transplantation may have an anti-fibrotic effect by suppressing TGF-β-stimulated fibrotic gene production in lung fibroblast cells.

### 2.3. Stem Cell-Derived Mitochondria Demonstrated an Excellent Anti-Fibrotic Effect

The cellular sources for mitochondrial transplantation may be tissues, cell lines, primary cells, and stem cells. We then investigated the anti-fibrotic effect of the mitochondria isolated from different cellular sources. Supplementary Figure S1 shows that mitochondria prepared from UC-MSCs, but not from HEK293, CHO, and L6 cells, induced pluripotent cells (iPSCs), and exhibited the suppression of TGF-β-stimulated collagen expression in lung fibroblast cells.

**Figure 3:**
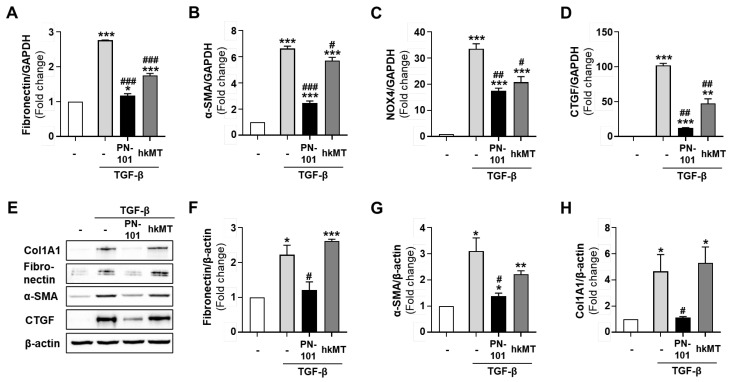
Anti-fibrotic effects of stem cell-derived mitochondria. Anti-fibrotic effects of UC-MSC-derived mitochondria (PN-101) were compared with those of HEK293-derived mitochondria (hkMT). (**A**–**D**) The mRNA expression of fibrotic genes was assessed by qRT-PCR. PN-101 mitigated the mRNA expression of fibronectin, α -SMA, NOX4, and CTGF in TGF-β-stimulated CCD8-Lu cells, whereas hkMT did not. (**E**–**H**) Col1A1, fibronectin, α-SMA, and CTGF protein levels were assessed with a Western blot assay. PN-101, but not hkMT, attenuated fibrotic protein expression in TGF-β-stimulated CCD8-Lu cells. * *p* < 0.05, ** *p* < 0.01, and *** *p* < 0.001 vs. control. ^#^ *p* < 0.05, ^##^ *p* < 0.01, and ^###^ *p* < 0.001 vs. mitochondria non-treatment.

We evaluated the expression of various fibrotic genes in the TGF-β-treated CCD8-Lu cells, followed by PN-101 and hKMT treatment, to further compare the anti-fibrotic effect between PN-101 and mitochondria (hkMT) isolated from HEK293 cells. Quantitative RT-PCR results demonstrated that PN-101 reduced the mRNA expression of fibronectin, α-SMA, NOX4, and CTGF induced by the TGF-β much more than hkMT (Figure 3A–D). A similar effect was also observed in the Western blot assay. PN-101 significantly abolished fibronectin, α-SMA, Col1A1, and CTGF protein expression stimulated by TGF-β treatment in lung fibroblasts, whereas hkMT did not (Figure 3E–H).

### 2.4. PN-101 Suppressed TGF-β-Induced Mesenchymal Phenotype in HBECs

**Figure 4:**
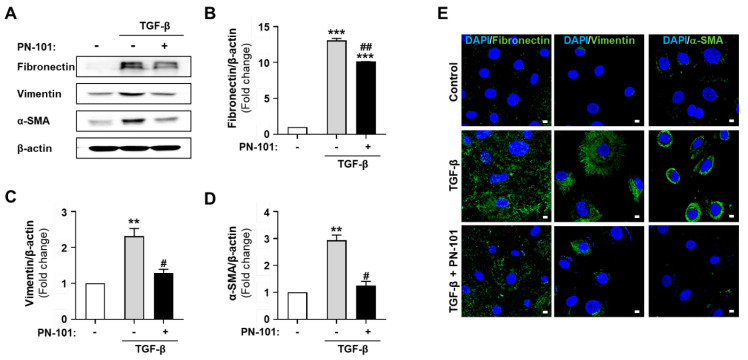
Epithelial-to-mesenchymal transition suppression by PN-101 in human bronchial epithelial cells. Human bronchial epithelial cells (HBECs) were treated with TGF-β of 2.5 ng/mL for 6 h after PN-101 administration for 24 h. (**A**–**D**) Fibronectin, vimentin, and α-SMA protein levels were evaluated by a Western blot assay. PN-101 attenuated fibronectin, vimentin, and α-SMA protein expression in TGF-β-stimulated HBECs. (**E**) An ICC assay demonstrated that PN-101 suppressed TGF-β-stimulated fibronectin, vimentin, and α -SMA protein expression in HBECs. The scale bar indicates 10 μm. Data are presented as the mean ± SEM (n = 3). ** *p* < 0.01 and *** *p* < 0.001 vs. control. ^#^ *p* < 0.05, and ^##^ *p* < 0.01 vs. PN-101 non-treatment.

EMT is a process of converting epithelial cells into mesenchymal cells and has a crucial role in PF. Additionally, TGF-β induces EMT in pulmonary epithelial cells, causing a substantial increase of myofibroblasts in PF [31]. The gene expression of mesenchymal markers (fibronectin and vimentin) and a myofibroblast marker (α-SMA) were analyzed in HBECs to investigate whether PN-101 has any effect on TGF-β-induced EMT in HBECs. The expression of fibronectin, vimentin, and α-SMA was significantly increased after TGF-β treatment compared with the control. Notably, PN-101 treatment alleviated the TGF-β-induced upregulation in mesenchymal and myofibroblast marker expression (Figure 4A–D). Similarly, the immunocytochemistry results confirmed the upregulation of fibronectin, vimentin, and α-SMA expression after TGF-β treatment compared with the control, and TGF-β-induced change in the expression was reversed by PN-101 treatment (Figure 4E).

### 2.5. Effects of PN-101 on Fibrotic Processes in Bleomycin-Induced PF Mice

**Figure 5:**
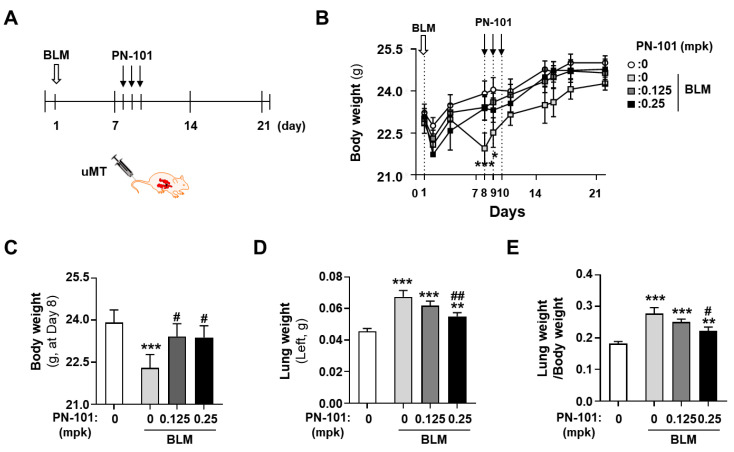
Effect of mitochondrial transplantation on body weight and lung weight. (**A**) Schematic representation of PN-101 administration to BLM-induced mice. The mice were intratracheally instilled with BLM of 0.33 mpk and intravenously injected three times with 2.5-μg PN-101/head (0.125 mpk) or 5-μg PN-101/head (0.25 mpk) 7 days after the BLM treatment. (**B**,**C**) BLM instillation decreased body weight and was restored to normal with PN-101 treatment. (**D**,**E**) BLM treatment increased lung wet weight even when expressed relative to body weight. * *p* < 0.05, ** *p* < 0.01, and *** *p* < 0.001 versus sham. ^#^ *p* < 0.05 and ^##^ *p* < 0.01 vs. PN-101 non-treatment.

We used bleomycin (BLM)-induced mice as an in vivo PF animal model [32]. Mice were administered with BLM intratracheally, and, 7 days later, were intravenously treated with PN-101 of 0.125 mg/kg (mpk) or PN-101 of 0.25 mpk for 3 consecutive days, as described in the schematic diagram, to evaluate the anti-fibrotic effect of PN-101 in a therapeutic window context (Figure 5A). A total of 32 mice were randomly allocated into four groups of 5 mice/group; sham, BLM, BLM + 0.125-mpk PN-101, and BLM + 0.25-mpk PN-101. One mouse in the BLM + 0.25 mpk group died of serious lung fibrotic pathology on day 10 after the intratracheal instillation of BLM.

The body weight, lung weight, and the ratio of lung weight to body weight were measured for monitoring the lung injury of BLM-injured mice [33]. As expected, intratracheal BLM instillation significantly decreased the body weight of the mice. Treatment with PN-101 of 0.25 mpk notably recovered BLM-induced body weight loss 8 days after BLM administration. Meanwhile, PN-101 of 0.125 mpk tended to prevent the BLM-induced body weight loss although it was statistically insignificant (Figure 5B,C). The lung weight was significantly increased approximately two-fold in the BLM group and was increased when expressed as lung weight per body weight. PN-101 treatment at 0.25 mpk significantly alleviated BLM-induced lung weight and the ratio of lung weight to body weight increased (Figure 5D,E). Similarly, PN-101 of 0.125 mpk tended to decrease the BLM-induced lung weight gain, although statistically not significant.

**Figure 6:**
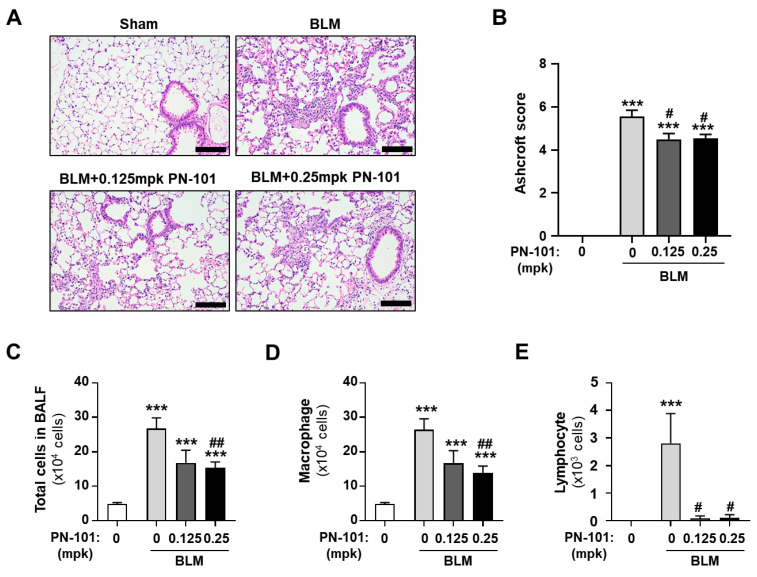
Attenuation of fibrotic processes by mitochondrial transplantation in BLM-induced PF mice. (**A**) Representative lung tissue sections stained with H&E. The sections in the sham group exhibited normal morphology, whereas those of the BLM group demonstrated structural disorder and alveolar septum widening. PN-101 treatment significantly improved these histopathological lesions. Scale bars indicate 100 μm. (**B**) The Ashcroft fibrosis score demonstrated that PN-101 significantly alleviated PF in BLM-treated mice. (**C**) The effect of PN-101 on total cell counts in the BALF of the BLM-induced PF mice. (**D**,**E**) The effect of PN-101 on the number of macrophages and lymphocytes in the BALF. Data are presented as the mean ± SD (n = 5). *** *p* < 0.001 vs. sham group. ^#^ *p* < 0.05 and ^##^ *p* < 0.01 vs. PN-101 non-treatment.

The histopathological changes in the lung were evaluated by performing H&E staining to investigate the in vivo effect of mitochondrial transplantation in the BLM-induced PF mouse model. Figure 6A shows that the BLM group demonstrated BLM-induced inflammatory and fibrotic processes. In contrast, PN-101 treatment of 0.125 mpk and 0.25 mpk into BLM-induced PF mice significantly exhibited reduced collagen fiber deposition and less alveolar wall destruction in the lung tissue sections. The Ashcroft score quantifying the histological changes in the BLM-treated mice demonstrated that PN-101-treated mice developed less severe fibrotic lesions compared with the BLM mice (Figure 6B).

We then assessed the number of total cells and all major leukocytes in the BALF. The total number of cells, macrophages, and lymphocytes both in the BLM group and the BLM + PN-101 group were significantly increased as compared with those in the sham group. However, PN-101 treatment of 0.25 mpk significantly attenuated the increase of total cells and macrophages, whereas PN-101 of 0.125 mpk demonstrated the tendency of increase without significance. Both PN-101 of 0.125 mpk and 0.25 mpk dramatically alleviated the BLM-induced increase of lymphocytes (Figure 6C–E).

## 3. Discussion

This study revealed that mitochondrial transplantation reduced fibrotic processes in in vitro and in vivo PF models. The results indicated that the exogenously isolated mitochondria were successfully transferred into the lung fibroblasts and bronchial endothelial cells. Mitochondria isolated from stem cells, but not from the immortalized kidney cell lines, reduced mRNA and protein expression of the extracellular matrix, which were closely related to fibrosis. Additionally, mitochondrial transplantation suppressed the EMT in the pulmonary epithelial cells. Lastly, the intravenously administered mitochondria protect against bleomycin-induced lung injury through the modulation of fibrotic processes in the IPF mouse model.

Mitochondria are crucial for cell survival by providing ATP as the powerhouse via oxidative phosphorylation. Additionally, they play a nonbioenergetic role in controlling the balance of reactive oxygen species (ROS) and inflammatory response. Accumulating evidence has indicated that IPF pathogenesis is closely related to mitochondrial abnormalities, including reduced ATP production, impaired oxidative phosphorylation, excessive ROS production, incomplete mitophagy, and increased apoptosis [7,8,34]. Oxidative stress with high mitochondrial ROS levels and endogenous TGF-β signaling activation induces the EMT of alveolar epithelial cells, thereby aggravating lung fibrosis. Bioenergetic dysfunction with decreased ATP production linked to the rate of oxygen consumption and reduced mitochondrial membrane potential has also been found in primary IPF fibroblasts and an experimental model of lung fibrosis [35,36]. Impaired mitophagy and mitochondrial DNA were observed in IPF lung tissues [37,38,39]. Furthermore, NADPH oxidase 4 (Nox4), which is overexpressed in IPF lung fibroblasts, promotes fibroblast differentiation to myofibroblasts, in which mitochondrial biogenesis is downregulated [40]. Therefore, mitochondrial injury repair has been proposed as a novel approach for IPF treatment. Therefore, the beneficial effects of metformin, which improves mitochondrial function and biogenesis, have been observed in IPF [41]. Our current data demonstrated that TGF-β induced a decrease in ATP and MtMP levels in both lung fibroblasts and epithelial cells. Intriguingly, PN-101 treatment significantly mitigated the TGF-β-induced decrease in ATP and MtMP levels, indicating that mitochondrial transplantation may prevent PF development by reversing the mitochondrial abnormalities.

IPF is characterized not only by collagen accumulation and EMC deposition by activated fibroblasts (i.e., myofibroblasts) but also by severe lung structure distortion, ultimately causing respiratory failure [42]. Myofibroblasts as master cells in fibrosis express α-SMA and induce the expression of type I collagen and other ECM macromolecules, which distinguish them from typical fibroblasts [43]. Indeed, TGF-β as the key fibrogenesis regulator increases the number of alveolar epithelia cell-derived myofibroblasts by activating the EMT process [44,45,46]. Further, the TGF-β pathway promotes alveolar fibroblast differentiation into myofibroblasts, which are the primary producers of pro-fibrotic genes, including ECM components, thereby developing fibrosis by the excessive ECM deposition in tissues. Therefore, targeting the excessive ECM accumulation would be a potential option for fibrosis treatment. Hence, our current data demonstrated that TGF-β significantly increased the expression of col1 A1, fibronectin, and α-SMA in human lung fibroblasts, and mitochondrial transplantation dramatically reverted TGF-β-induced EMC expression. Moreover, mitochondrial transplantation suppressed the EMT process in lung epithelial cells by decreasing fibronectin, vimentin, and α-SMA expression, which act as important EMT markers. Together, mitochondrial transplantation would attenuate lung fibrosis by inhibiting myofibroblast formation via fibroblast and EMT differentiation of epithelial cells, ultimately causing the suppression of ECM accumulation.

Mitochondria for mitochondrial transplantation studies may be derived from diverse cell types or tissues, including cardiomyocytes, mesenchymal stem cells, platelets, hepatocytes, and adipocytes [47,48,49,50,51]. Syngeneic, allogeneic, and xenogeneic mitochondria isolated from different cellular sources have demonstrated beneficial effects and uptake efficacy in various in vitro and in vivo disease models. The current study compared the anti-fibrotic activity of stem cell-derived mitochondria with that of nonstem cell-derived mitochondria. Stem cell-derived mitochondria suppressed TGF-β-induced collagen deposition in the lung fibroblast cells, whereas nonstem cell-derived mitochondria did not. The superiority of stem cell-derived mitochondria in terms of efficacy in the in vitro fibrosis model over nonstem cell-derived mitochondria remains unclear, although both mitochondria have similar capacities in ATP production and cellular uptake efficacy [52]. Recent studies have demonstrated that stem cells donate their mitochondria to injured cells as well as take up damaged mitochondria from injured cells through tunneling nanotubes [52,53,54,55]. This spontaneous mitochondrial transfer from stem cells rather than somatic cells provides impaired cells with cellular function improvement, including proliferation, antiapoptotic function, cellular metabolism and respiration, and oxidative stress. Recently, cell therapy with MSCs, where mitochondria were enriched by pioglitazone treatment, demonstrated the delay of fibrotic progression in a fibrosis model [56]. Therefore, stem cell-derived mitochondria may be the best candidate as a therapeutic agent to treat IPF with mitochondrial dysfunction.

Currently, the Food and Drug Administration and the European Medicines Agency have approved nintedanib and pirfenidone as treatment drugs for IPF [2,3]. Both drugs have delayed disease progression and mitigated the aggravation of acute IPF, but they are unable to halt disease progression and repair lung damage. Additionally, they have limitations for treatment options due to adverse effects [57]. Consequently, a critical medical unmet need is to develop novel therapeutic agents to overcome these limitations. We observed that the intravenously injected mitochondria mitigated the lung damage and decreased the inflammatory cells of the BALF in the therapeutic mode in the in vivo BLM-induced IPF mouse model. Our studies revealed that mitochondrial transplantation improved the fibrotic environment by suppressing lung fibroblast and EMT activation of lung epithelial cells, but it remains unclear if mitochondrial transplantation alleviates the mitochondrial damage in IPF. Therefore, whether mitochondrial transplantation improves mitochondrial biogenesis, oxygen consumption, and intracellular ROS generation in in vitro and in vivo IPF models need to be determined. Although the effectiveness of PN-101 transplantation was shown in BLM-induced IPF mice, there is a limitation in that no single animal model is not enough to provide complete outcomes because of the highly complicated pathobiology of IPF. Therefore, future studies would be necessary that the efficacy of PN-101 for IPF treatment should be investigated using various IPF animal models including intratracheal instillation of silica or asbestos, radiation, and transgenic mice.

Despite the limitation, to the best of our knowledge, this is the first report demonstrating that exogenous mitochondrial transplantation mitigates the fibrotic process in a BLM-induced animal model.

In conclusion, defects in mitochondrial function and myofibroblast activation were observed in the IPF model and were successfully rescued by mitochondrial transplantation. Mitochondrial transplantation is a therapeutic intervention as it suppresses ECM deposition in lung fibroblasts and EMT in lung epithelial cells. Mitochondrial transplantation may provide a promising new treatment option for IPF.

## 4. Materials and Methods

### 4.1. Cell Culture

Human lung fibroblasts (CCD8-Lu) and human bronchial epithelial cells (HBECs) were purchased from ATCC (Cat no. CCL-201, Manassas, VA, USA) and PromoCell (PromoCell, Heidelberg, Germany), respectively. Human umbilical cord-derived mesenchymal stem cells (UC-MSC) were obtained by primary culture of umbilical cord from a healthy pregnant woman with informed consent. This study was approved by Public Institutional Review Board (IRB) designated by the Ministry of Health and Welfare, Republic of Korea (IRB No. P01-202002-31-008). Human induced pluripotent stem cells (iPSCs) and human embryonic kidney cells (HEK-293) were kindly provided Dr. Baek Soo Han (Korea Research Institute of Bioscience and Biotechnology) and Dr. Chang Hwan Park (Hanyang University), respectively. CCD8-Lu cells were cultured in Dulbecco’s modified Eagle’s medium (DMEM, cytiva, Marlborough, MA, USA) supplemented with 10% fetal bovine serum (FBS) and antibiotics (100 units/mL of penicillin and 100 μg/mL of streptomycin) at 37 °C in a 5% CO_2_ atmosphere. HBECs were cultured in an Airway Epithelial Cell Growth Medium supplemented with SupplementMix (PromoCell, Germany) at 37 °C in a 5% CO_2_ atmosphere. UC-MSCs were grown in Minimum Essential Medium Eagle Alpha Modification (α-MEM; Hyclone, Logan, UT, USA) supplemented with 10% FBS and basic fibroblast growth factor (FGF-2; CHA Meditech Co., Daejeon, Republic of Korea) of 10 ng/mL at 37 °C in a 5% CO_2_ atmosphere. iPSCs were seeded on matrigel-coated (Invitrogen, Waltham, MA, USA) plates and were fed with Essential 8 Flex Medium (Invitrogen) according to the manufacturer’s protocol. HEK-293 cells were cultured in DMEM supplemented with 10% Fetal Bovine Serum (FBS) and antibiotics (100 units/mL of penicillin and 100 μg/mL of streptomycin) at 37 °C in a 5% CO_2_ atmosphere.

### 4.2. Isolation of Mitochondria from PN-101 and hkMT

The mitochondria were obtained from the UC-MSCs and HEK-293 by differential centrifugation, as previously reported [22], and referred to as PN-101 and hkMT, respectively. Briefly, the cells were disrupted with nitrogen cavitation and then centrifuged at 2000× *g* for 10 min at 4 °C. The supernatant was further centrifuged at 12,000× *g* for 15 min to precipitate the mitochondria. The pellet was suspended in 500-μL of SHE buffer (20 mM of HEPES, 250 mM of sucrose, 2 mM of EGTA, pH of 7.4) and then centrifuged at 20,000× *g* for 10 min. The final pellet was resuspended in 100-μL of SHE buffer and kept at 4 °C. The quantity of mitochondria was identified by a bicinchoninic acid (BCA) assay.

### 4.3. Labeling of the Mitochondria

The mitochondrial targeting sequence (MTS) of the succinate dehydrogenase complex subunit C was fused with the red fluorescent protein (dsRED), causing MTS-dsRED. Lentiviral expression vectors were constructed by cloning the MTS-dsRED genes into a lentiviral vector plasmid with EF1α promoter. The HEK293T cells were seeded on 10-cm dishes at 70–80% confluency for lentivirus production. The cells were then cotransfected with 4 μg of lentiviral expression vector plasmid along with 4 μg of pLP1, 4 μg of pLP2, and 3 μg of pLP/VCVG (Packaging Mix, Invitrogen). The transfection mixtures were prepared in 1 mL of Opti-MEM containing DNA and 30 μL of 293Tran^TM^ (Origene, Rockville, MD, USA) following the manufacturer’s protocol. The lentiviral supernatant samples were collected 24, 48, and 72 h after transfection. The collected lentiviral supernatants were concentrated with polyethylene glycol 6000 and suspended in an α-MEM medium. UC-MSCs were seeded into 150-mm plates at 70–80% density and then incubated with lentiviral supernatants and 4 μg/mL of polybrene, followed by further incubation for 72 h, to label the mitochondria with dsRED.

### 4.4. ATP Assay

CCD8-Lu cells and HBECs were plated 0.8 × 10^4^ in 96-well plates and cultured overnight. The existing media was removed and replaced with serum-free DMEM overnight. TGF-β of 2.5 ng/mL was then added. 8 µg of PN-101 was added overnight after 6 h to remove TGF-β media of 2.5ng/mL. CellTiter-Glo^®^ reagent (Promega, Madison, WI, USA) of 100 μL prepared at room temperature was added to each well to remove the existing media. The luminescent signal was measured every 2.5 s for 20 min after shaking the plate on a plate reader for 30 s.

### 4.5. MtMP Assay

CCD8-Lu cells and HBECs were plated 2 × 10^5^ in 6-well plates and cultured overnight. The existing media was removed and replaced with serum-free DMEM overnight. TGF-β of 2.5 ng/mL was then added. After 6 h, 20 µg of PN-101 was added overnight to remove TGF-β media of 2.5 ng/mL. The cells were incubated with TMRE of 500 nM at RT for 10 min and analyzed by flow cytometry.

### 4.6. Quantitative Reverse Transcription-Polymerase Chain Reaction (RT-PCR) Assay

CCD8-Lu cells were plated 2 × 10^5^ in 6-well plates and cultured for 24 h. Cells were cultured with serum-free DMEM for a further 24 h. TGF-β of 2.5 ng/mL was added to induce the fibrotic process. After 6 h, the cells were treated with 20 µg of PN-101 or hkMT for 18 h. Total RNA was isolated from the cells with the TRIzol reagent (Life Technologies, Carlsbad, CA, USA, 15596026). The synthesis of complementary DNA (cDNA) was performed with an M-MLV cDNA synthesis kit (Enzynomics, Daejeon, Republic of Korea). Quantitative real-time PCR analysis was conducted with the MIC magnetic induction cycler (BioMoleculer Systems, Seoul, Republic of Korea) using a 2xSYBR Green (Phile Korea, Seoul, Republic of Korea). The specific primer sequences include human GAPDH antisense, 5′- ACC ACC CTG TTG CTG TAG CCA A -3′, human GAPDH sense, 5′- GTC TCC TCT GAC TTC AAC AGC G -3′ and human fibronectin antisense, 5′- GTG GCT GAA GAC ACA AGG AA -3′, human fibronectin sense, 5′- CCT GCC ATT GTA GGT GAA T -3′ and human α-SMA antisense, 5′- TCC CAC CAT CAC CCC CTG ATG TC -3′, human α-SMA sense, 5′- GCC CAG CCAA GC ACT GTC AGG A -3′ and human CTGF sense, 5′- GGC TTA CCG ACT GGA AGA C -3′, human CTGF antisense, 5′- AGG AGG VGT TGT CAT TGG -3′ and human NOX4 Antisense, 5′- GGC TCT GCT TAG ACA CAA TCC -3′, human NOX4 Sense, 5′- CAC CTC TGC CTG TTC ATC TG -3′. The activation comprised 95 °C for 2 min and cycling conditions comprised 95 °C for 10 s and 60 °C for 20 s. The value of each gene expression was normalized to that of GAPDH.

### 4.7. Western Blotting Assay

CCD8-Lu cells and HBECs were plated 2 × 10^5^ in 6-well plates and cultured for 24 h. Cells were cultured with serum-free DMEM for a further 24 h. TGF-β of 2.5 ng/mL was added to induce the fibrotic process. After 6 h, the cells were treated with 20 µg of PN-101 or hkMT for 18 h. Cells were washed twice with phosphate-buffered saline (PBS), suspended in the lysis buffer (50 mM of Tris, 150 mM of NaCl, 1% NP-40, 0.5% deoxycholic acid, and 0.1% SDS), and disrupted by sonication. The protein samples were subjected to 15% SDS-polyacrylamide gel electrophoresis (PAGE) and transferred onto a nitrocellulose membrane (Sigma, St. Louis, MO, USA). The membrane was then incubated in 5% skimmed milk for blocking and then incubated with rabbit polyclonal anti-Col1A1 (Invitrogen, PA5-29569, 1:1000), mouse monoclonal anti-fibronectin (Santa Cruz Biotechnology, Dallas, TX, USA, sc-8422, 1:1000), mouse monoclonal anti-α-SMA (Sigma, A5228, 1:1000), rabbit polyclonal anti-CTGF (Invitrogen, PA5-32193, 1:1000), mouse monoclonal anti-vimentin (Santa Cruz Biotechnology, Dallas, TX, USA, sc-6260, 1:1000), and mouse monoclonal anti-β-actin (Sigma, A2228, 1:5000) at 4 °C for 12 h with gentle shaking. The secondary antibodies, including goat anti-rabbit IgG HRP or anti-mouse IgG HRP, were utilized. The protein expression images were obtained with an enhanced chemiluminescent substrate (Sigma, St. Louis, MO, USA).

### 4.8. Immunocytochemistry

CCD8-Lu cells were plated 2 × 10^5^ in 6-well plates and cultured for 24 h. Cells were cultured with serum-free DMEM for a further 24 h. To induce the fibrotic process, 2.5 ng/mL of TGF-β was added. After 6 h, the cells were treated with 20 µg of PN-101 or hkMT for 18 h. Cells were fixed in 4% formaldehyde for 30 min, washed with 1 PBS twice, and then incubated with blocking buffer (PBS containing 10% goat serum and 0.2% Triton X-100) for 2 h. The cells were then incubated overnight at 4 °C with a primary antibody (rabbit polyclonal anti-Col1A1; 1:1000, mouse monoclonal anti-fibronectin; 1:1000, mouse monoclonal anti-α-SMA 1:1000 and mouse monoclonal anti-vimentin 1:1000) in PBS containing 1% normal goat serum and 0.1% Triton X-100. Appropriate fluorescence-tagged secondary antibodies (Jackson Immunoresearch Laboratories, West Grove, PA, USA) were used to achieve visualization after PBS washes. The stained samples were mounted in VECTASHIELD with DAPI mounting solution and photographed with the Olympus confocal microscope (FV3000, Olympus, Tokyo, Japan).

### 4.9. Animals and Mitochondrial Treatment

Seven-week-old male C57BL/6 mice were purchased from Orient Bio (Seongnam-si, Republic of Korea). The animals were randomly assigned to the following four experimental groups, five mice per group: (1) sham, (2) BLM, (3) BLM + 2.5 μg/head of PN-101; and (4) BLM + 5 μg/head of PN-101. The mice received a single intratracheal instillation of 0.33 mg/kg of BLM in 50-μL of saline using an automatic video instillator to establish the IPF mouse model [58]. The BLM dose was selected based on a preliminary study of a BLM-induced IPF mouse model. PN-101 was intravenously injected once daily from day 8 to day 10 after the BLM treatment. All animals were necropsied 3 weeks after the BLM treatment and were euthanized by nitrous oxide inhalation with 5% isoflurane and humanely sacrificed by exsanguination from the vena cava and aorta. This study was approved by the Institutional Animal Care and Use Committee of the Korea Institute of Toxicology (IACUC # IAC-24-01-0176-0117; approval date: 17 June 2024).

### 4.10. Body Weight and Lung Weight

The body weight of each mouse was measured before the first treatment and on days 2, 4, 8, 9, 11, 15, 16 and 18. The terminal body weights were measured on the termination day (day 22). The left lung was weighed to identify the absolute and relative weights based on the terminal body weight ratios.

### 4.11. Bronchoalveolar Lavage Fluid (BALF) Analysis

The BALF preparation was collected with PBS of 0.7 mL three times in the right lung. The total number of cells in the BALF was quantified and measured with a NucleoCounter (NC-250; Chemometec, Gydevang, Denmark). The cells in the BALF were then subjected to Diff-Quik staining (Sysmex, Kobe, Japan) for differential cell counting under a light microscope.

### 4.12. Histopathological Examination

After weighing the left lung, it was fixed in a 10% neutral-buffered formalin solution over 24 h, embedded in paraffin, and sectioned into 2 μm thick slices. Lung sections were prepared and stained with hematoxylin and eosin (H&E) for histopathological examination. Histopathological analysis was conducted with a light microscope at 200× magnification (scale bars represent 100 μm). Lung fibrosis scores were evaluated using a modified Ashcroft scoring system and assessed on a scale of 0–8 [59].

### 4.13. Statistical Analysis

Data are presented as the mean ± standard error of the mean (SEM). All statistical analyses were conducted with GraphPad Prism version 8.0.1 (GraphPad Software Inc., San Diego, CA, USA). A one-way analysis of variance with Dunnett’s post hoc test was performed for multiple comparisons.

## 5. Conclusions

This study revealed that the transplantation of mitochondria isolated from human mesenchymal stem cells alleviated pulmonary fibrosis by inhibiting lung fibroblast or EMT activation of lung epithelial cells. Moreover, the intravenous administration of mitochondria suppressed the fibrotic process in BLM-induced IPF mice. Thus, these results indicate that mitochondria could serve as a promising novel modality for IPF treatment.

## Data Availability

The data collected and analyzed during the current study are available from the corresponding author upon reasonable request.

## Supplementary Materials

The supporting information can be downloaded at: https://www.mdpi.com/article/10.3390/ijms252312783/s1.
