# Supplementary material for: Mitochondrial Transplantation Ameliorates Pulmonary Fibrosis by Suppressing Myofibroblast Activation
Source: Int J Mol Sci. 2024 Nov 28;25(23):12783. doi: 10.3390/ijms252312783 (PMC11641484; doi:10.3390/ijms252312783)
Supplement: Supplementary file 1 [file ijms-25-12783-s001.zip › ijms-3327841-supplementary.pdf]

Article

Mitochondrial transplantation ameliorates pulmonary fibrosis by suppressing myofibroblast activation

Seo-Eun Lee [1,†], Shin-Hye Yu [1,2,†], In-Hyeon Kim [3,4], Young Cheol Kang [1], Yujin Kim [1], Jeong Seon Yeo [1], Jun Hyeok Lim [1], Iksun Kwon [1], Je-Hein Kim [3], Se-Woong Park [3,4], Mi-Yoon Chang [2,5], Kyuboem Han [1], Sung-Hwan Kim [3,*] and Chun-Hyung Kim [1,*]

[1] Paean Biotechnology, Inc., 5 Samil-daero 8-gil, Jung-gu, Seoul 04552, Republic of Korea
[2] Graduate School of Biomedical Science and Engineering, Hanyang University, Seoul 04763, Republic of Korea
[3] Division of Jeonbuk Advanced Bio Research, Korea Institute of Toxicology, Jeongeup 56212, Republic of Korea
[4] College of Veterinary Medicine, Chonnam National University, Gwangju 61186, Republic of Korea
[5] Department of Premedicine, College of Medicine, Hanyang University, Seoul 04763, Republic of Korea
* Correspondence: sunghwan.kim@kitox.re.kr (S.-H.K.); chkim@paeanbio.com (C.-H.K.)
† These authors contributed equally to this work.

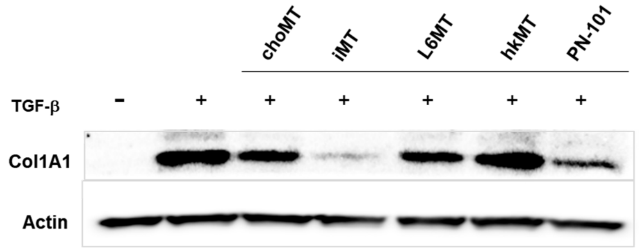

Supplementary Figure S1. Stem cell-derived mitochondria exhibited an excellent anti-fibrotic effect. Mitochondria were isolated from UC-MSCs, iPSCs, HEK293, CHO, and L6 cells, respectively, and were then treated with TGF-β-stimulated CCD8-Lu cells. Stem cell-derived mitochondria suppressed TGF-β-induced collagen expression in the lung fibroblast cells, whereas somatic cell-derived mitochondria did not.
